# Extraordinary Nanocrystalline Pb Whisker Growth from Bi-Mg-Pb Pools in Aluminum Alloy 6026 Moderated through Oriented Attachment

**DOI:** 10.3390/nano11071842

**Published:** 2021-07-16

**Authors:** Matic Jovičević-Klug, Patricia Jovičević-Klug, Tina Sever, Darja Feizpour, Bojan Podgornik

**Affiliations:** 1 Institute of Metals and Technology, Lepi pot 11, 1000 Ljubljana, Slovenia; patricia.jovicevicklug@imt.si (P.J.-K.); tina.sever@imt.si (T.S.); darja.feizpour@imt.si (D.F.); bojan.podgornik@imt.si (B.P.); 2 International Postgraduate School Jožef Stefan, Jamova cesta 39, 1000 Ljubljana, Slovenia

**Keywords:** nanocrystalline nanostructure, crystal growth, nanorods, aluminum alloy, metallic whisker, internal stress, oriented attachment

## Abstract

The elucidation of spontaneous growth of metal whiskers from metal surfaces is still ongoing, with the mainstream research conducted on Sn whiskers. This work reports on the discovery of Pb whisker growth from Bi-Mg-Pb solid pools found in common machinable aluminum alloy. The whiskers and hillocks display unique morphologies and complex growth that have not been documented beforehand. In contrast to typical understanding of whisker growth, the presented Pb whiskers show a clear nanocrystalline induced growth mechanism, which is a novel concept. Furthermore, the investigated whiskers are also found to be completely composed of nanocrystals throughout their entire length. The performed research gives new insight into nucleation and growth of metal whiskers, which raises new theoretical questions and challenges current theories of spontaneous metal whisker growth. Additionally, this work provides the first microscopic confirmation of recrystallization growth theory of whiskers that relates to oriented attachment of nanocrystals formed within an amorphous metallic matrix. The impact of mechanical stress, generated through Bi oxidation within the pools, is theoretically discussed with relation to the observed whisker and hillock growth. The newly discovered nanocrystalline growth provides a new step towards understanding spontaneous metal whisker growth and possibility of developing nanostructures for potential usage in sensing and electronics applications.

## 1. Introduction

Metal whisker formation has been extensively researched within the last 50 years as they are the major cause of electrical shorts, and thus failure of electronic devices [1]. The mainstream of research of the phenomena, although existing in many metals and alloys, has been focused on the Sn metal and Sn alloys, since a significant fraction of electronic devices are constructed from these materials [1]. Despite the tremendous research effort, the theory of whisker growth is still not clear. As a result, many proposed nucleation and growth mechanisms exist for the explanation of whisker formation. The most commonly accepted theories are the residual stress induced growth [2,3,4] and the intermetallic compound (IMC) growth [5]. Both rely on the presence of internal stress, which induces growth by the dislocation loop slip governed by the Bardeen–Herring source mechanism and local diffusion of atoms and vacancies that relax local stress gradients [6,7]. However, several researchers have also provided results that contradict these theories and discussed that other mechanisms are potentially involved in the formation of metal whiskers (see comprehensive review by Zhang et al. [1]). One of these mechanisms is the recrystallization theory, which was firstly proposed by Ellis et al. [8] and was also one of the theories that disproved the prior dislocation driven whisker growth theories designed by Frank [9] and Eshelby [10]. The theory provides the necessary explanation for development of whiskers from any metallic structure by the development of a new surface-bound crystal that exerts a whisker growth once the surrounding material agglomerates sufficient strain energy to cause motion of dislocations and material along the principal axis of the newly developed grain. The theory is especially interesting from the standpoint of the initial spontaneous nucleation and growth of the whiskers, which is considerably faster than at a later stage [11] and is unexplainable by the traditional diffusion-based theories [12]. At the moment the main issue of this theory is the lack of direct experimental evidence for such a mechanism, since the theory has been proven only through indirect evidence. The recrystallization theory has been updated with relation to the dynamic recrystallization effect proposed by Vianco and Rejent [13,14], which has been experimentally confirmed [14,15]. However, this theory is specific for thermally cycled material and provides no explanation of whisker formation in materials, where such material processing is not performed. Another theory that explains the rapid development and growth of whiskers is the fluid flow mechanism [16,17], which is based on creep development process, which provides concrete results on the development of whisker under different stress and thermal states [16]. The theory also connects the relation to the cracking oxide theory and the internal stress state of the material [18]. Nevertheless, the theory has been mainly associated with experiments dealing with externally stressed materials and provides still limited explanation when the whiskers form under ultra-high vacuum conditions and when the original material lacks the specific crystalline columnar structure and internal stresses. From the literature review and comprehensive and critical reviews of Zhang et al. [1] and Jagtap et al. [19], it is clear that the conducted research and developed theories do not provide a clear consensus about the exact whisker nucleation and growth model that would reliably predict the whiskering phenomenon in different metallic materials and surfaces under different conditions. Furthermore, it seems that the individual theories are applicable to an extent and that the theories have to be selected on a case-to-case basis rather than applying a single theory and model to explain all the whiskering phenomenon observed on a variety of materials and conditions.

Despite the main interest of whisker research in Sn-containing alloys, the whiskering phenomenon is not exclusive to Sn and its alloys. Researchers have reported the formation of whiskers from other metals, such as Au [20], Al [21], Bi [22], Cd [23], Pd [24], Zn [25], etc. Such whiskers also display similar characteristics as those found in Sn alloys and are generally observed on coatings or bulk materials that have been exposed to either elevated temperatures, external stresses or surface damage processes such as abrasion [11,26]. The main accepted concept for formation of such whiskers is also mainly associated to stress induced whisker growth caused by intermetallic phases, residual stresses developed during material preparation or externally applied stress. This also holds for Pb whiskers [27,28], which have been only scarcely researched and have been mainly researched in connection with Sn alloys [11,29,30,31,32]. Research of naturally occurring and spontaneously grown Pb whiskers at room temperature outside of Sn-containing alloys has not been performed until now.

In this research, the discovery of spontaneous growth of Pb whiskers in a metallic system formed within a conventional lead-containing aluminum alloy EN AW 6026 is disclosed. Aluminum alloy EN AW 6026 is a recently developed alloy, which is commonly used as a substitute for EN AW 6082 [33], EN AW 6081 [33], EN AW 2011 [34] and EN AW 2030 [34] in industry for decorative and hard anodizing, hot forged and machined parts. The alloy is also specifically used for development of electrical and electronic parts and automotive components in cars, train (high-speed train [35]) brake systems and robotics/prosthetics [34,36,37]. The alloy is referentially used, due to its high corrosion resistance and moderate resistance behavior in high stress/high temperature application, which is a result of its limited tin content (up to 0.05 wt.%). Sn normally induces weakening and cracking in other aluminum alloys. Since the material is widely applied in sensitive components, the investigation and understanding of the temporal surface behavior is crucial, which led to the discovery of Pb whisker growth in EN AW 6026. This first-time discovery of Pb whiskers in such a material and in general, such detailed analysis of spontaneously grown and naturally occurring Pb whiskers has previously not been performed. The material is also interesting as the generation of Pb whiskers is induced within specific Bi-Mg-Pb regions, which results in growth of whiskers and hillocks with uncommon morphologies. Furthermore, these uncommon growths indicate a potential novel method for production of nanoparticles and nano-structures without complex and expensive deposition techniques. Finally, the work reports on nanocrystalline growth of the whisker, which also occasionally translates into a nanocrystalline growth of the entire whisker. Such behavior has not been recorded until now for spontaneously grown metal whiskers and gives the first direct experimental evidence of the recrystallization induced whisker growth mechanism proposed by Ellis et al. [8].

## 2. Materials and Methods

Cuboid samples of dimensions 55 mm × 10 mm × 10 mm were machined from the commercial EN AW 6026 alloy rods provided by Impol, Slovenia. The exact chemical composition of the investigated alloy, which was determined with inductively coupled plasma-optical emission spectrometry (ICP-OES) with Agilent 720 (Agilent, Santa Clara, CA, USA), is provided in Table 1. The samples were heat treated with a standard procedure (homogenization) performed at 570 °C for 1 h and afterwards quenched in water. Subsequently, the samples were cut, polished and their surface finished with colloidal silica (25–40 nm particle size, 15 N load, 3 min polishing time). The observation of whiskers on the samples’ surfaces was performed with scanning electron microscopy (JEOL JSM-6500F (JEOL, Tokyo, Japan) and Zeiss Crossbeam 550 (Carl Zeiss AG, Oberkochen, Germany)) and light microscopy (Zeiss Axio Imager.Z2m, Carl Zeiss AG, Oberkochen, Germany) immediately after metallographic polishing and 24 h, 7 days, 14 days and 21 days after sample preparation. The samples were stored during the measurements in a dry storage space at room temperature. For statistical relevance, 6 samples were monitored with the same measurement scheme described above. The scanning electron microscopy was conducted with an acceleration voltage of 15 kV and a working distance of 10 mm. The angled imaging with SEM was performed with an observation angle of 70° at a working distance of 19 mm. Chemical composition was analyzed by energy-dispersive X-ray spectroscopy (Oxford EDS INCA Energy 450 (Oxford Instruments, Abingdon, UK), detector type INCA X-SIGHT LN2 (Oxford Instruments, Abingdon, UK)).

Zeiss CrossBeam 550 system was also used to cut the whiskers and underlying material with focused Ga+ ion beam (FIB). The sample was tilted under 54° and the cuts in the material were made normal with respect to the 0° reference plane. Firstly, a 1 μm thick protection layer of platinum was deposited on the surface of the chosen features. Afterwards, a trench of the size of 10 × 10 × 10 μm^3^ was etched with Ga+ ion beam at accelerating voltage 30 kV and current 30 nA, decreasing in multiple steps to 300 pA for a final polish of the cross-section. The sample was inspected after every step of the milling by secondary electrons at 5 kV and current 2 nA, and Ga+ beam at 30 kV and 50 pA.

For transmission electron microscopy (TEM) and scanning TEM (STEM) analysis, the whiskers were scooped up from the sample surface after 21 days after surface preparation and placed on a Cu 200 mesh lacey carbon TEM grid. TEM was carried out in a JEOL JEM-2100HR microscope (JEOL, Tokyo, Japan) at 200 kV to determine morphology, crystalline structure and elemental composition of the whiskers. For TEM imaging and electron diffractions the Gatan Orius SC 1000 CCD camera (Pleasanton, CA, USA) was used and for elemental composition TEM or STEM with bright-field (BF) and dark-field (DF) detectors were used, combined with energy dispersive X-ray spectroscopy (EDS) with JED-2300 (JEOL, Tokyo, Japan) for point and line analyses, and elemental mappings. Elemental mappings were recorded from around 30 min to almost 2 h, depending on the area of the analysis and analysis conditions.

## 3. Results and Discussion

### 3.1. Microstructure and General Morphology

**Figure 1:**
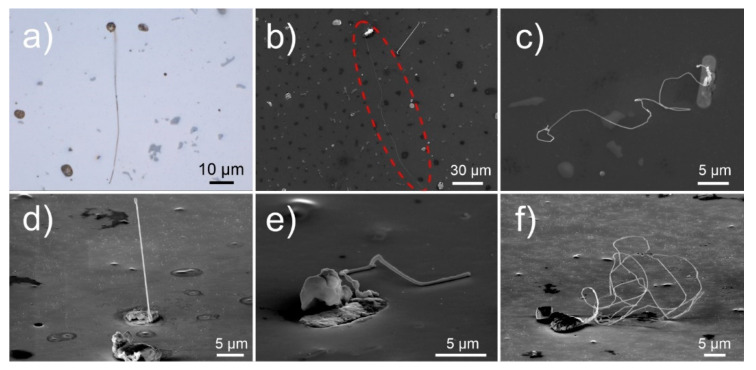
Micrographs of Pb whiskers obtained with (**a**) light microscopy, and (**b**–**f**) scanning electron microscopy (secondary electron imaging). Images (**d**,**e**) are obtained by tilting the sample by 70°.

The microscopic observation of whiskers was performed periodically after the surface of the aluminum alloy (see experimental section for material preparation) was metallographically polished, in order to observe the time-dependent whisker growth and development. After 24 h, long whiskers (length exceeding 50 µm) form from the Bi and Pb-containing solid regions (see Figure 1a–c). These regions will be further denoted as pools, due to their specific rounded shape and clear separated structure from the aluminum matrix. The pools are scattered across the sample with an average density of about five pools per 10,000 µm^2^, from which on average two pools demonstrate whisker growth (Figure 1a). The whiskers grow to considerably large lengths, with one of the longest found to be roughly 160 µm (Figure 1b—marked with dashed red oval), corresponding to an average growth rate of 0.6 nm/s within the first 24 h. The whiskers can grow in disfigured forms with the growth orientation changing across the length as can be seen in Figure 1c. With a side view of the longer whiskers, it is also clear that the disfiguration does not occur consistently. The whiskers display the common perpendicular growth from the pool surfaces as seen in Figure 1d. However, the growth in many cases is diverted, and the thickness of the whiskers varies across the length (Figure 1e), leading to complex intertwined whiskers with large cumulative lengths (Figure 1f).

**Figure 2:**
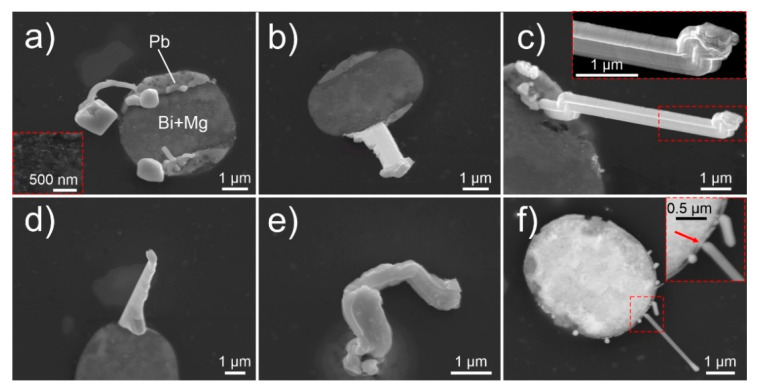
(**a**–**f**) Micrographs of various Pb whiskers and hillocks obtained with scanning electron microscopy. Images (**a**–**e**) are obtained with secondary electron imaging, image (**f**) is obtained with backscattering electron imaging. The inserts in figures (**c**,**f**) display the enlarged areas marked by dashed red box. The red arrow in insert of figure (**f**) indicates the narrow region of Pb zone of the Bi-Mg-Pb pool.

The whiskers mainly grow from the edges of the pools, which is a result of the internal structure of the pools that consist of two defining regions. The inner piece consists of Bi incorporating Mg with atomic fraction from 5% to 45%. The combination of both elements suggests a double phase system constructed of Bi_2_Mg_3_ and Bi, which was also observed clearly in certain pools (see insert in Figure 2a). With energy-dispersive X-ray spectroscopy (EDS), presence of oxygen was detected with atomic fraction of 2/3x to 1x the atomic fraction of Bi, suggesting that most of the upper part of Bi is in oxide form (see supporting Figure S1). The outer part of the pool from which the whiskers grow (between Bi region and the aluminum matrix) consists of pure Pb (marked in Figure 2a) covered by an oxide (PbO) layer. The whiskers are confirmed to be mainly composed of Pb at which occasionally Bi is present in them in the form of solid solution (5–18 at.%), when the whiskers grow from or near the Pb–Bi interface. The whisker growth is induced by the compressive stresses built by a combination of the quenching process of aluminum alloy from the high temperature as well as by the different solidification temperatures and different thermal expansion coefficients of the matrix and individual pool regions [38,39]. The whisker growth from the pools results in the fascinating morphologies, which to the authors’ knowledge have previously not been reported for whiskers and other spontaneous growth of crystals from metal surfaces. The occurrence of the unique whisker and hillock morphologies have been identified to occur considerably often, since from 1300 observed whiskers and hillocks, around 700 displayed the unique morphologies that are discussed through representative examples in the following sections.

### 3.2. Temporal Whisker Evolution

**Figure 3:**
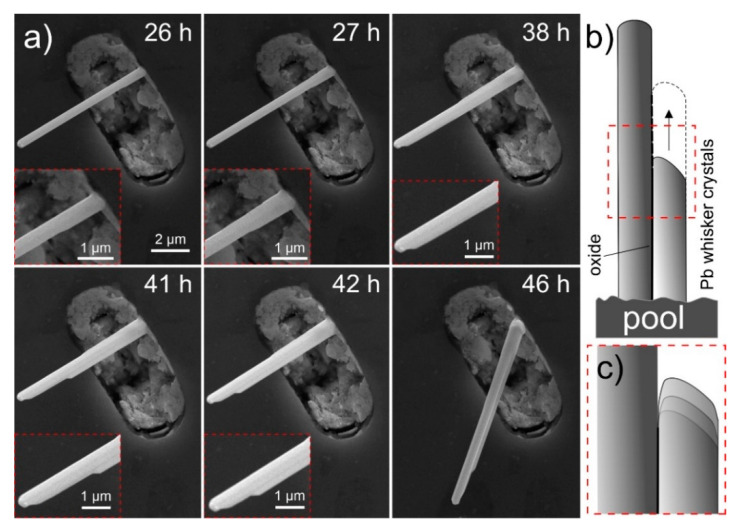
(**a**) Time evolution of whisker widening through growth of additionally activated facets at the side of the main crystal. The individual inserts portray enlargements of sites of interest for comparison of individual images. The timestamp denotes the time from the final polishing of the surface. (**b**) Schematic representation of stepwise whisker growth with (**c**) enlarged portion displaying the incremental growth that corresponds to the serrations found in individual whisker crystals as seen in Figure 2c.

In specific cases, hillocks form as bulk faceted crystallites, underneath which the considerably thinner whiskers grow (Figure 2a). In contrast to the regular forming hillocks (Figure 2d), the faceted hillocks (Figure 2a,b) are already on their own an unexpected feature, especially since these growths contradict the regular whisker growth theories [3,5,40,41,42,43] that do not predict the surface-parallel widening growth into faceted crystals. Furthermore, these growths indicate that either the whiskers grow from a perpendicularly oriented crystal underneath the hillock or the hillock over time reduces its width due to the reduced stress and/or driving force. The later explanation, however, is dismayed by other whiskers that display stepwise widening of the whisker with the additional activated crystal growth as seen in Figure 2b. Both formations suggest that the mechanism of whisker growth is considerably more complex as explained by the traditional theories of low angle surface grains [3,40]. Another complex morphology is displayed in Figure 2c, where a fourling crystal structure emerges equally from the pool. The crystals jointly follow every change in growth direction and are of similar diameter. The growth serrations seen on the front-facing two crystals clearly depict the connected growth as well as the twinned structure seen by the mirror angle tilt of growth serrations with respect to the grain boundary between the two crystals (see insert of Figure 2c). Bundled whisker growth has been reported in the past [41], however such fourling crystal growth is a unique event that displays that the growth of whiskers can be highly ordered from multiple conjoined grains and supports the theory of recrystallization induced whisker growth [13,14]. To further emphasize the bundled growth and stepwise widening of the whiskers by stepwise activation of individual crystal facets, a series of images is provided in Figure 3 displaying the temporal growth of an individual bundled whisker within 20 h. The figure clearly displays the growth of whiskers is not governed by an individual crystal as proposed by traditional whisker growth theories [3,5,40,41,42,43]. The series of images also clearly visualize the fast-pace growth of the individual widening segments (compare image at 41 h and 42 h) displaying 1.3 µm growth within 1 h, which corresponds to an average growth rate of 0.36 nm/s. The series also disclosed a clear growth development from bottom-up direction, as the top and side of the whisker do not change with growth (see individual image inserts of Figure 3). The enlargements of individual segments also unveil that the individual side-grown crystals seem to fuse with the original main whisker with a thin oxide between them (schematically presented in Figure 3b,c). Such behavior suggests crystal growth mechanics related to oriented attachment phenomena [44,45,46,47], which suggests a low diffusion barrier and a nanocrystalline structure of the individual crystal segments.

**Figure 4:**
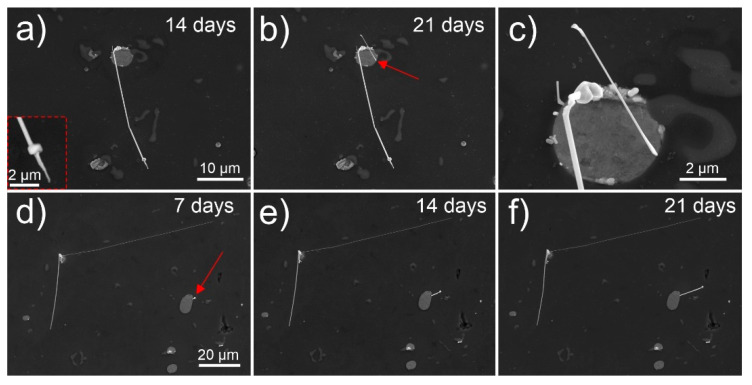
Scanning electron microscopy micrographs (obtained with secondary electron imaging) of Pb whisker growth with (**a**–**c**) spontaneous growth and with (**d**–**f**) continuous growth. Image (**c**) is a magnified view of the region of Bi-Mg-Pb pool in image (**b**) with the same timestamp of image (**b**). The red arrows indicate the positions of interest for the observation of whisker growth. The timestamp denotes the time from the final polishing of the surface.

The whisker growth is not only limited to completely opened pools. As seen in Figure 2e, the whiskers also grow from small opening of the pools that barely accommodate sufficient area for the whisker formation. As seen with Sn whiskers [5,41,48,49], the surrounding material constricts the whisker growth, which results in a fluted surface of the whisker and twisted whisker form. The most unexpected growth of whiskers, which is probably connected with the same growth mechanism as seen for faceted hillocks, is the growth of thin whiskers from a narrow lower region of the whisker (see Figure 2f). The whisker seems to grow from a single narrow region of a Pb ring (marked by red arrow in insert of Figure 2f) in an outward faceted form. The highly unusual growth has been observed in only limited number of pools and only for whiskers with thickness in the range of up to about 200 nm. The possible explanation for the unusual growth possibly lies in the ratio of residual stress to the thin Pb region and the limited quantity of available material for the whisker growth. It is possible, that the nucleation of whiskers is induced by recrystallization of the surface of the Pb region, which results in the formation of a nano-sized seed crystal, which acts as a defining element for the orientation and growth of the whisker. The seed crystal can potentially grow preferentially before the formation of a whisker, which results in the formation of a faceted hillock. Once the whisker starts to form underneath the hillock and the underlying whisker builds a critical pressure, the hillock is detached from the sample surface. The resulting growth anomaly can be seen in Figure 4a that shows a long whisker with a narrow tip, followed by a wider region and further followed by a longer whisker growth with an intermediate width.

The whiskers discussed so far show that the growth process of single whiskers is mainly activated within the first 24 h from the preparation of the surface. Nevertheless, growth of additional whiskers was determined to form also several days later, which has also been reported for Sn whiskers [2,5,30,40]. Within this research, the growth of whiskers was observed to vary significantly. Some whiskers display no signs of prior slow formation of a hillock and display a sudden spontaneous whisker growth as has been observed for individual whiskers grown in the first 24 h (see Figure 4a–c). For the presented example, the formed thin whisker (marked by red arrow) forms from the edge, where no hillock is observed beforehand. In contrast, other whiskers display continuous growth over several days as seen in Figure 4d–f (the whisker position is marked by red arrow). Additionally, the preexisting long whiskers seem to stagnate across a long period. The different growth rates are presumably connected with the width of the whisker as well as by the position of its growth and the width of the Pb region within the pool. However, a detailed continuous statistical evaluation of the growth rates needs to be conducted in order to confirm such statement.

**Figure 5:**
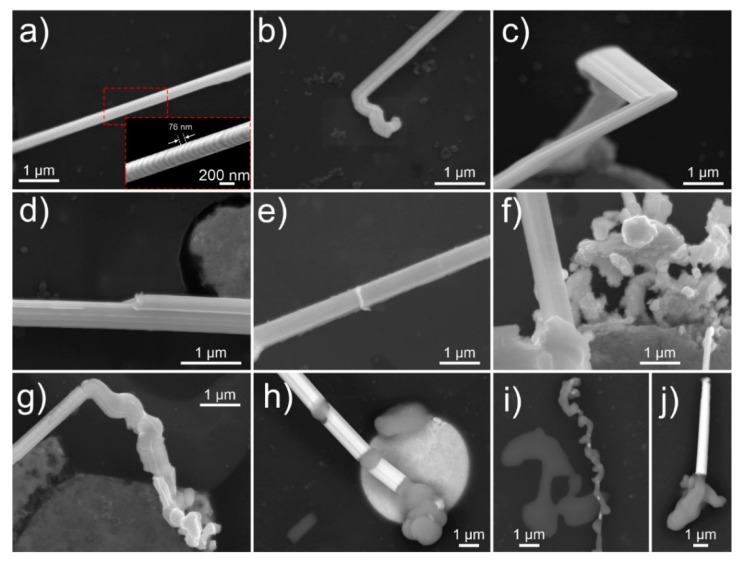
(**a**–**j**) Scanning electron microscopy micrographs of Pb whisker segments indicating the various observed growth phenomena. Images (**a**–**g**) are obtained with secondary electron imaging, image (**h**–**j**) are obtained with backscattering electron imaging. The insert in figure (**a**) displays the enlarged areas marked by dashed red box.

The varying growth dynamics are also further observed with higher magnification of individual whisker segments. The serrated growth of the individual whiskers, as seen beforehand, is visible also for the single crystal whiskers (Figure 5a). The serrations are considerably thin, in the range of 50–80 nm in width (see insert of Figure 5a), which correspond to individual single crystal growth segments that interchange in orientation. The origin of the segmentation is either from the slight shifting of individual slip planes [50,51] and nano-twinning [52] due to pipe diffusion caused by the stacking fault energy [19] or segmentation caused by repeatedly breaking and reforming of the surrounding oxide layer at the whisker base [3]. The growth of whiskers has been also seen to change throughout the growth process. Formations of caps (Figure 5j) and curved initializations of whiskers (Figure 5b) are present for all whiskers, which will be discussed in more detail with transmission electron microscopy (TEM) observations. The whiskers also display changing direction of growth (Figure 2c and Figure 5c), correlating to the preferred crystal orientation during growth [43]. Oxides also have a defining influence on the whisker morphology [53], which induce fluting (Figure 5c) and stepwise widening of whiskers (Figure 5d). The whiskers also accommodate an oxide layer on their surface, which can be seldom seen as scales on the surface of the whisker (Figure 5e). When the whiskers enter a stage of retarded growth, which is considered to occur due to the relaxed stress and/or material depletion, the ending part of the whiskers grows in a dysmorphic manner (Figure 5g). This section of the whisker morphologically resembles the curved initialization of the whisker, which suggest that the formation of such morphology is potentially connected with the growth speed. In rare occasions, after 28 days of surface preparation, specifically altered form of whiskers with different chemical composition is also observed. As can be seen in Figure 5h–j, the individual regions are contrasted light gray. The EDS of the regions (see supporting Figure S1) indicate a high amount of C (50% and upwards), which suggests that a reaction (most probably some form of catalytic) occurs between the Pb and native carbon layer and/or carbon contamination on top of the sample, when the retarded growth occurs. However, more research is needed to understand these formations and the reaction that governs such modification of the whisker structure.

### 3.3. Nanocrystalline Structure

**Figure 6:**
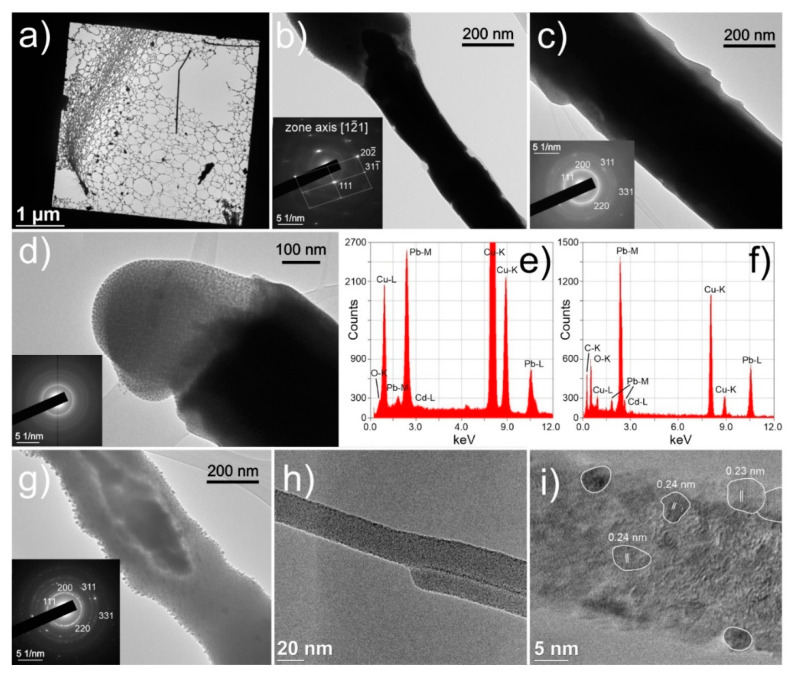
(**a**–**d**) and (**g**–**i**) Transmission electron microscopy images of sections of Pb whiskers. The inserts in images (**b**–**d**) represent the individual selective area electron diffraction patterns obtained from the center part of the corresponding images. Energy dispersive X-ray spectrograms of (**e**) central, bulk part of Pb whisker and (**f**) side region of Pb whisker with oxide layer. The Cu signal originates from the Cu gratings of the holding grid. In (**i**) the resolving power is limited by the thermal drifting of the sample, resulting in isolated focused regions/crystals for which the interatomic distances are determined. The marked interatomic distances correspond to the {200} of the Fm-3m Pb.

The different whisker structures are investigated on a nanoscale level using TEM to provide more insight into the different morphologies and formation of Pb whiskers collected from the sample surface (Figure 6a). As can be seen in Figure 6b, the middle and denser parts of the whiskers are in many cases constructed of single crystalline Fm-3m Pb (COD card 1011119, ICDD card 00-001-0995), which was confirmed with selective area electron diffraction (SAED) (insert of Figure 6b) and EDS (Figure 6e). The bulk segments also display widening and narrowing parts of the single crystal formations with intermediate breaks in-between them (Figure 6b), which correlates to the serrations observed for many whiskers (see Figure 5a). Furthermore, such morphology indicates that the construction of the microscopic whisker is from larger nanocrystals fused together at their ends along the same orientation, which further supports the claim of the presence of oriented attachment process during the whisker growth. In contrast to the bulk of whiskers, the whiskers’ extremities and ends display a nanocrystalline structure within an amorphous base as seen in Figure 6c,d. The extremities are additionally covered with an oxide layer PbO, which was determined with EDS (Figure 6f). The whiskers’ caps (Figure 6d) are confirmed to be completely nanocrystalline embedded within an amorphous matrix (SAED in insert of Figure 6d) at which only minute amount of oxygen was determined for these regions. Furthermore, individual whiskers and whisker segments also display nanocrystalline structure throughout their entire length (Figure 6c,h). Individual whiskers were also overexposed to the electron beam, causing local fusion of the nanocrystals and amorphous phase into a monocrystal (Figure 6g), which supplementary confirms the multicrystalline Pb structure as determined from the SAED (insert in Figure 6g). The nanocrystalline growth is also observed for extremely thin nano-sized whiskers as seen in Figure 6h. These whiskers were also analyzed with higher magnifications, which displayed that the oxide layer is extremely thin and that the whisker is composed of differently oriented Pb nanocrystals of different sizes and orientation (see Figure 6i). The chemical composition and whisker construct were also confirmed with scanning TEM (STEM), the results of which are presented in the supporting Figure S2.

The full-length growth of whiskers in a nanocrystalline form indicates that the mechanism for growth of whiskers results from the recrystallization of Pb material in the pool. A possible explanation is that the compressive stress is reduced through the formation of new crystals as has been proposed by the recrystallization theory first proposed by Ellis et al. [8] and later refined by other authors [13,14]. The recrystallization explains the initial curved growth, as the surrounding oxide and the varying local stress gradient molds the form of the abutted nanocrystals in varying direction and width. Furthermore, the recrystallization theory also accords with the abnormal whisker growth from the single narrow region (Figure 2f) and the growth of faceted hillocks (Figure 2a). The inclusion into the recrystallization theory is the apparent relation of the preferential growth of a single crystal during large growth speeds, which relates to the high influx of material that stabilizes the quasi-single crystal growth. As the influx of the material is reduced with the growth of the whisker and the reduction of stress, the quasi-single crystal growth is replaced by the nondirectional growth/movement of nanocrystals, resulting in the formation of non-oriented bulky structures.

### 3.4. Oxidation Effect

**Figure 7:**
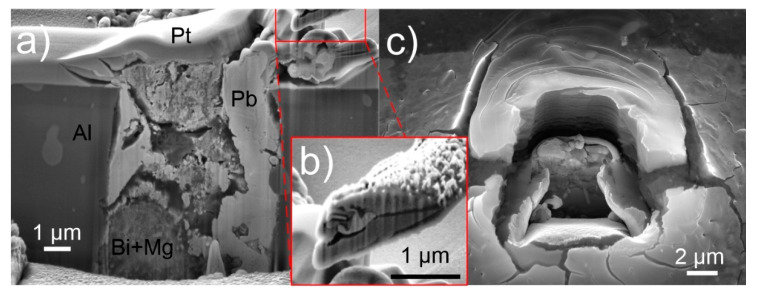
(**a**) Cross-sectional view of a Pb-Bi-Mg pool covered with Pt protection layer. The individual regions of the cross-section are marked with their main elemental composition. (**b**) Cross-sectional view of Pb whisker grown from pool presented in (**a**). (**c**) Image of a focused ion beam milled trench after exposure for 24 h in ambient environment showing a stress-induced fracture formation.

Further explanation can be provided by the cross-sectional investigation of the pool and whisker, which was conducted with focused ion beam (FIB) milling and SEM imaging. The resulting profile provided in Figure 7a indicates that the pools show separation regions of Bi-Mg and Pb. However, the Bi-Mg regions are in many cases intermixed with pockets of Pb, indicating a possible amorphous or glassy structure of the pool. The cross-section also reveals that bulk fused Pb regions exist on the sides of the pool. These regions are correlated with the growth of whiskers, as the whisker grew from the extended portion of the right-hand region of the pool. The identical whisker was also milled with FIB to acquire a cross-sectional view of the whisker structure. The cross-section, presented in Figure 7c, confirms the whisker composition of multiple subsets of elongated single crystals that grow parallel from the pool Pb section. The inner portion of the whisker shows a clear separation of the individual crystals with a thin layer of material, which is presumed to be lead oxide. The central crystal, considered to be the first and leading whisker crystal, displays a different crystallographic orientation compared to the surrounding crystals, which have a similar crystal orientation between each other. A similar construct for tin whiskers has been also observed by Courey et al. [49,54], but has not been related to recrystallization effect or step-wise growth of individual crystals. The EDS analysis of the cross-section confirms no presence of any specific intermetallic compounds or crystals that could induce the whisker growth as observed for tin whiskers with IMC [5]. The Bi-Mg regions of the pool exhibit a porous structure, which suggests that the possible reason for whisker formation could be related to the oxidation of the Bi-Mg region through diffusion of oxygen into the porous structure. This is further supported by the formed stress-induced fracturing of the surrounding aluminum matrix upon exposure of the FIB milled trench to ambient environment for 24 h (Figure 7c). The delamination and the large cracking suggest a significant build-up of stress and supports the theory of stress induced whisker formation caused by oxidation of the Pb-Bi-Mg pool.

**Figure 8:**
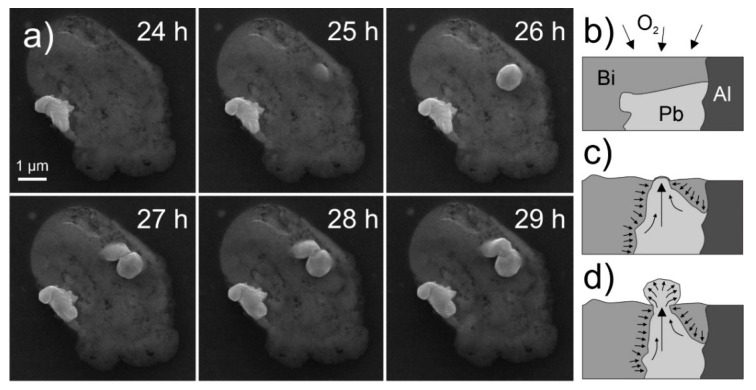
(**a**) Sequential images displaying hillocks growth formed from Pb regions situated underneath the Bi-Mg material of the pool. The timestamp denotes the time from the final sample surface preparation. (**b**–**d**) Schematic representation of the formation of facetted-bubble hillocks from a Bi-Mg-Pb pool with underlying Pb region. The arrows in (**b**,**c**) represent the movement of the material and the pressure state of the individual material segments.

This effect was tested by producing an additional sample, and after preparation, the sample was immediately stored in vacuum in the SEM and observed continuously. Within the first 24 h, only minute formation of hillocks occurred without the development of any pure whisker formation as observed beforehand. Afterwards, the same sample was further exposed to ambient room environment for 24 h and observed once more with SEM. Most of the pools developed whiskers as seen beforehand (Figure 2). However, individual pools exhibited a massive increase in number of hillocks with limited growth and negligible formation of whiskers. The growth pattern and the size of the individual miniature hillocks correspond to the mixed region found in the cross-sections of the pools as seen in Figure 7a, thus giving further support to the oxidation theory of Bi-Mg. An example series of images is given in supporting Figure S3. Such pools also give clear indication that the emergence of hillocks and later whiskers comes from specific Pb portions of the pools and that the portions do not have to be directly exposed to the external environment. A set of images presented in Figure 8a presents the emergence of spherical hillocks from the surface of the pool under which a Pb region is situated. The two independently formed hillocks show the different formations that are considered to be caused by the different form of the underlying Pb region. A graphical representation of the growth phenomenon is given in Figure 8b–d. It is presumed that the oxidized Bi-Mg regions (Figure 8b) exert a hydrostatic pressure onto the Pb regions that are compressed and deformed (Figure 8c). Once the Pb region reaches the surface, the Pb material expands outwards in a bubble form as the high pressure allows the expansion in all directions (Figure 8d). The hillocks reveal over time (Figure 8a) remolding of their form and a faceted form, which correlates with the oriented attachment theory proposed beforehand.

### 3.5. Theoretical Evaluation

The results indicate a complex mechanism behind the whisker growth that induces not only the formation of hillocks and whiskers, but also allows their reforming. The combination of oriented attachment and recrystallization are considered to be the only clear explanation for such growth mechanism. The predisposition for such a mechanism is the amorphous form of the Pb regions in the pools, which is possible due to the fast quenching of the sample material. The quenching should deliver quenching rates as large as 150 °C/s [55] that fall in the range of amorphization of pure metals [56,57]. Such a state of the material was confirmed by performing electron backscatter diffraction on the Pb regions, which yielded no Kikuchi pattern. With the increased pressure onto the Pb regions by the oxidized Bi-Mg regions of the pools, the Pb region develops random nucleation and formation of nanocrystals. Such development is known to occur for amorphous metals and requires large pressures in the GPa range [58,59]. However, individual amorphous particles of iron have shown a significantly lower melting temperature at applied pressures in the 100 kPa range [60]. With the high pressures determined from this study that should reach well over the proof strength of the aluminum matrix (330 MPa [33,34]), the reduction of the crystallization temperature may fall close to the room temperature values [61], which would allow the formation of nanocrystals. The growth of nuclei is considered to follow the non-classical driven nucleation as has been observed for amorphous Bi [62]. However, the nanocrystals form in a limited manner as the densification (causing change in volume Δ*V* and elastic energy *E* contribution) through crystallization leads to reduction of pressure *P*, which induces an increase of the Gibbs free energy nucleation barrier Δ*G* and limited diameter *d* as indicated by Equation (1) [59]:

(1) $$\Delta G\left(T,P\right)=\pi d^{3}\left(\Delta G_{v}+E\right)/6+\pi d^{2}\gamma +P\Delta V$$

**Figure 9:**
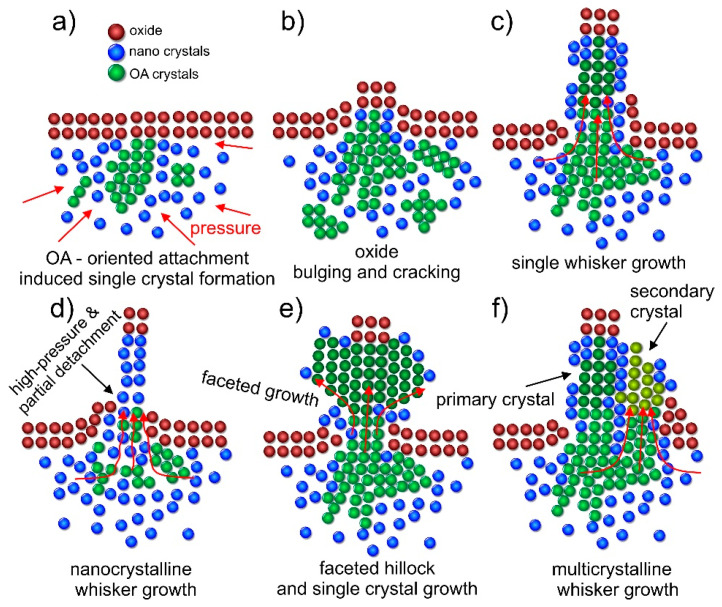
Schematic representation of the different crystallization growths caused by recrystallization and oriented attachment in the Bi-Mg-Pb pools. For clarity, the amorphous portion of the Pb material is not presented. (**a**) presents the agglomeration of nanocrystals at specific positions with their rearrangement formed by oriented attachment. (**b**) presents the bulging of the underlying material from the material’s surface that occurs due to cracking of the overlying oxide layer. The subsequent sketches present the growth and development of (**c**) a single-crystalline whisker, (**d**) nanocrystalline whisker, (**e**) faceted single-crystalline hillock and (**f**) multicrystalline whisker with subsequent secondary crystal growth.

Based on the volumetrically unconstricted nucleation and energy dependency of the nuclei (volume energy *G_v_* and surface interface energy between crystalline and amorphous phase *γ*), the nanocrystals are of spherical form, which is confirmed from the TEM observations (Figure 6). As the size limitation of the nanocrystals is also formed by the pressure itself, as it limits the atomic motion [60], a portion of the Pb material remains amorphous. With the pressure and constriction of the Pb regions, the Pb material behaves like a fluid with the nanocrystals “floating” within the amorphous media in a Brownian-like motion. With the local collisions and agglomerations of the nanocrystals, oriented attachment occurs (Figure 9a). This mechanism is then further enhanced with the local bulging and cracking of the oxide material or overlaying Bi-Mg material, which give way to the expansion of the Pb material outside of the material’s surface (Figure 9b) along specific crystallographic orientation that are most close-packed [16]. As the orifice is limited in size, it creates a bottleneck formation and localized increased pressure. The increased pressure allows further growth of new nanocrystals on a local scale and increased fusion of crystals through oriented attachment due to the locally increased imputed energy [46,62,63]. This is considered to be a crucial moment that decides the form of the whisker growth.

When the pressure is high, but allows gradual alignment of the nanocrystals along a preferential crystallographic orientation, the single whisker growth forms (Figure 9b). With the fusion and rearrangement of the nanocrystals [62] the general orientation of the whole whisker is conserved once a preferential alignment is set. However, the growth can occur in a discontinuous form, resulting in whisker segmentation (Figure 6b) as suggested by the serration formations on the whiskers (Figure 2c and Figure 5a). This is considered to be related to the detachment of the lower section of the growing whisker from the surrounding material (oxide layer and partial crystalized regions around the whisker). The detachment occurs when the critical pressure builds up, caused by the agglomerated crystallites and additional migration of the crystallites onto the surface of agglomerations and orifice. The latter also leads to the amorphous surface formed for the single crystal whiskers (Figure 6c). In the case of exceedingly large pressures caused by a small orifice, the nanocrystals still agglomerate, but cannot orient correctly due to the fast growth and high pressure that causes also partial detachment of the nanocrystals. The formed misoriented agglomeration and lack of general orientation of crystallites along a specific crystal orientation results in the growth of thin nanocrystalline whisker (Figure 9d). This mechanism also explains the formation of the disorganized whisker caps, as the crystallites on the top are exposed to exceeding pressure, which is imposed before the surface emergence. The misoriented and detached nanocrystallites are held together by the accompanying amorphous phase that acts as a binder. In the case of a larger orifice and lower pressure, the large hillocks form (Figure 9e), which can form facets depending on the predetermined crystal orientation and pressure at the neck of the hillock. It is proposed that the hillocks form preferentially, when an explosive growth occurs in the beginning, erupting large quantities of material. The large volume of the material then either forms into the dysmorphic growth with continuously high pressure or allows remolding of the hillocks into faceted forms that slowly grow when the pressure is reduced. The faceting is formed directly as a result of the oriented attachment and reorganization coupled with the minimization of the surface energy through facete formation, which is also observed and theoretically evaluated for Sn whiskers [64]. All the discussed growths can be of mixed type depending on the pressure changes, Pb region volume and the speed of Bi-Mg expansion, leading to coupled forms such as hillock-whisker form as observed in Figure 2a. The length limitation of the whiskers is considered to be related to the mass of the whisker and the pressure required to move the already exerted mass with the underlying reservoir of the material and pressure within the pool. These limitations can halt the whisker growth and result in the multicrystalline whisker growth that is formed by the preferential collapse or pushing of the oxide layer at the sides of the primary whisker crystal (Figure 9f). Regardless of the growth mechanism, the whiskers also include a portion of the amorphous Pb material that resides between the nanocrystals and the edges of the whiskers and hillocks. This correlates well with the bleak SAED rings observed also for whiskers that exhibit a single crystal form (Figure 6b,c).

### 3.6. Impact and Potential Applications

The disclosed growth mechanism and morphology of the whiskers and hillocks open many opportunities for the exploitation of the structures for both fundamental research of whisker growth as well as development of nanostructures for practical applications. The first major opportunity of the system is to disclose the relation of the possible presence of amorphous phase and nanocrystallites during the nucleation and growth of whiskers. Many researchers state that the formation of IMC [5,64] has a leading role in the development of whiskers and their growth causes the expansion of the whisker length and additional whisker growth over time [12]. However, the IMC growth has limited explanation for the exact origin of the preferential crystallite chosen for the whisker growth, which can be of different crystallographic orientation [65]. A recent study of Tian et al. [66] discloses that whiskers do not necessarily hold a grain boundary at the whisker root, which indicates that the generally accepted grain boundary diffusion [16] is not the only mechanism that governs the whisker formation. The discovered nanocrystallite formation might hold the key to explaining the whisker formation through diffusional growth and reformation of nanocrystallites, situated at the surface boundaries of larger crystallites. Furthermore, the presence of amorphous phase and spherical nanocrystallites has been recently proven in Sn coating [67], which further supports the possible presence of a nanocrystalline sub-structure of larger crystallites in Sn alloys as well as the formation of nanocrystalline structured whiskers.

The unique pool structures placed within the aluminum matrix and the relation to the oxidation of the Bi phase of the pool allows further research of the whisker growth with relation to external influencing factors such as oxidating media, intrinsic residual stress of the matrix and temperature. Such parametrization can lead to novel complex nanostructure formation that can be used for different applications. One of the most perspective structures is the core-shell feature of the single crystal whiskers with the amorphous and oxidated shell. Such structures have the potential to be utilized for gas sensing applications [68], hydrogen storage [69] as well as for various electronics applications [69,70]. Another possible application is development of self-regenerating aluminum surface covered with regrowing Pb whiskers for antimicrobial purposes [71]. The applicability of the Pb whiskers is even further expanded with the manipulation of the microrod surfaces through reactive chemical processes. The oxidized whisker surface can be employed for catalytic functionality [72], whereas with incorporation of doping elements and surface synthesis with C, S and N the development of semiconductor microrods is feasible for electronic applications [73,74]. The main still opened question of this system is the control and throughput of the whisker formation and growth, which, with further experimental work, can be explored and determined.

## 4. Conclusions

The newly discovered Pb whisker formed on EN AW 6026 aluminum alloy display growth from Bi-Mg-Pb pools with an average nucleation and growth formation of 2 whiskers per 10,000 µm^2^ in 24 h after final surface preparation. The whiskers display varying growth speeds, reaching lengths as long as 160 µm in a 24 h period. These pure Pb whiskers display stepwise growth activation as well as temporal widening of a preexisting whisker through additional facete activation, which is a significantly different growth behavior compared to the growth of conventionally found whiskers in Sn materials. Additionally, the combination of whisker and hillocks growth from a narrower opening and modification of the growth through the widening and narrowing of the whiskers is associated to a complex growth mechanism that results in unique whiskers and hillocks morphologies. The whisker growth mechanism and specific morphologies are elucidated to be related to a nanocrystalline structure formation of the original amorphous Pb material that is modified through pressure, formed by Bi oxidation. Some of the exerted whiskers also display a complete nanocrystalline structure across their entire length, which is a feature that has until now not been reported for spontaneously naturally occurring metallic whiskers. The whiskers can display both single and multicrystalline structure, which together with their unique growth behavior is explainable by the arrangement and fusion of the observed nanocrystals through oriented attachment, coalescence and surface migration. As such, these results support greatly the recrystallization theory of whisker nucleation, which explains many of the unique structures and morphologies of observed hillocks and whiskers. The additional mechanism of oriented attachment and nanocrystalline structure also allow the explanation of the whisker growth from a phase lacking grain boundaries and a defined crystalline structure, which are considered to be a general prerequisite for whisker formation in other metallic systems. The presented results and the proposed mechanism of inducing whisker growth through oxidation of a neighboring phase has the potential to elucidate the origin of the whisker growth and the varying nucleation time of whiskers in some metallic systems. The new system raises many questions about the theoretical understanding of whisker growth and nucleation, but also provides many new possibilities to understand the whisker evolution in more detail through the newly introduced concepts and mechanism. Finally, the system opens up a new opportunity to develop and tailor nanosized metallic micro- and nanorods and nanocrystallites for various potential applications through a simple process that does not require complicated and expensive procedures. The system and nanostructured formation will be further studied in more detail, with emphasis on manipulating and controlling the growth mechanism of Pb whiskers with varying external parameters such as pressure, temperature and oxidizing media.

## Figures and Tables

**Table 1:** Composition of investigated aluminum alloy AW EN 6026.

Element	wt.%
Al	balance
Mg	0.70
Si	0.68
Bi	0.66
Mn	0.59
Pb	0.34
Cu	0.30
Fe	0.27
Cr	0.045
Ti	0.029
Zn	0.025
Sn	<0.005

## Data Availability

The data presented in this study are available within the article itself and its supplementary material.

## Supplementary Materials

The following are available online at https://www.mdpi.com/article/10.3390/nano11071842/s1, Figure S1: Additional SEM images of Bi-Mg-Pb pools and whisker and corresponding EDS point measurements with table of elemental composition for each EDS point analysis. Figure S2: Additional STEM images and elemental mapping of selected whisker fragment. Figure S3: Additional SEM images of Bi-Mg-Pb pool exhibiting varying growth of hillocks based on the exposure to vacuum and ambient environment.
