# Supplementary material for: Extraordinary Nanocrystalline Pb Whisker Growth from Bi-Mg-Pb Pools in Aluminum Alloy 6026 Moderated through Oriented Attachment
Source: Nanomaterials (Basel). 2021 Jul 16;11(7):1842. doi: 10.3390/nano11071842 (PMC8308324; doi:10.3390/nano11071842)
Supplement: Supplementary file 1 [file nanomaterials-11-01842-s001.zip › nanomaterials-1244627-supplementary.pdf]

Supplementary Materials

Extraordinary Nanocrystalline Pb Whisker Growth from Bi-Mg-Pb Pools in Aluminum Alloy 6026 Moderated through Oriented Attachment

Matic Jovičević-Klug [1,*], Patricia Jovičević-Klug [1,2], Tina Sever [1], Darja Feizpour [1] and Bojan Podgornik [1,2]

1 Institute of Metals and Technology, Lepi pot 11, 1000 Ljubljana, Slovenia; patricia.jovicevicklug@imt.si (P.J.-K.); tina.sever@imt.si (T.S.); darja.feizpour@imt.si (D.F.); bojan.podgornik@imt.si (B.P.)
2 International Postgraduate School Jožef Stefan, Jamova cesta 39, 1000 Ljubljana, Slovenia
* Correspondence: matic.jovicevicklug@imt.si; Tel.: +386-1-4701-973

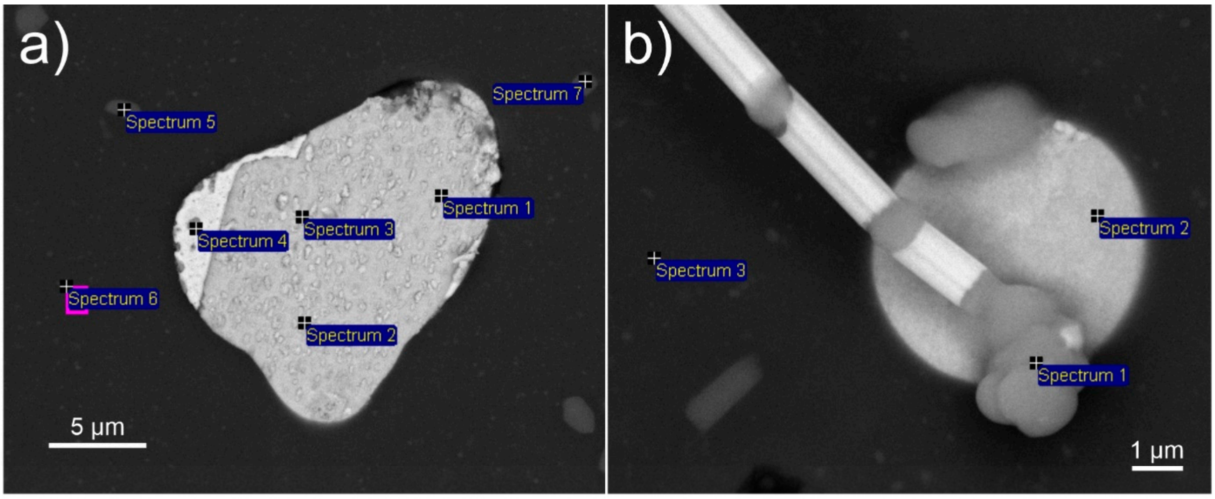

Spectrum	O	Mg	Al	Si	Mn	Fe	Cu	Pb	Bi
Spectrum 1	32.50	24.74	11.96	0.00	0.00	0.00	0.00	0.00	30.81
Spectrum 2	35.89	20.78	11.54	0.00	0.00	0.00	0.00	0.00	31.80
Spectrum 3	37.02	26.51	9.60	0.00	0.00	0.00	0.00	0.00	26.87
Spectrum 4	32.54	3.91	18.98	0.00	0.00	0.00	0.00	42.71	1.85
Spectrum 5	3.24	0.52	76.96	7.78	5.69	5.80	0.00	0.00	0.00
Spectrum 6	0.00	0.76	97.37	1.15	0.50	0.00	0.23	0.00	0.00
Spectrum 7	2.02	0.48	79.64	6.94	5.25	5.31	0.35	0.00	0.00

Spectrum	C	Mg	Al	Si	Pb	Bi
Spectrum 1	80.66	0.54	10.92	0.00	7.88	0.00
Spectrum 2	36.00	27.18	12.64	0.00	0.00	24.18
Spectrum 3	7.36	0.74	91.14	0.76	0.00	0.00

Figure S1. Scanning electron images acquired with back scattering electron imaging of a (**a**) Bi-Mg-Pb solid pool and a (**b**) solid pool exhibiting Pb whisker growth and formation of carbon enriched segments of the Pb whisker and Pb area. The individual points measured with energy-dispersive X-ray spectroscopy (EDS) are marked in each image. The quantitative evaluation of EDS spectra is given underneath each image in table form. The values in the tables represent the atomic fraction of individual elements for each acquired spectrum.

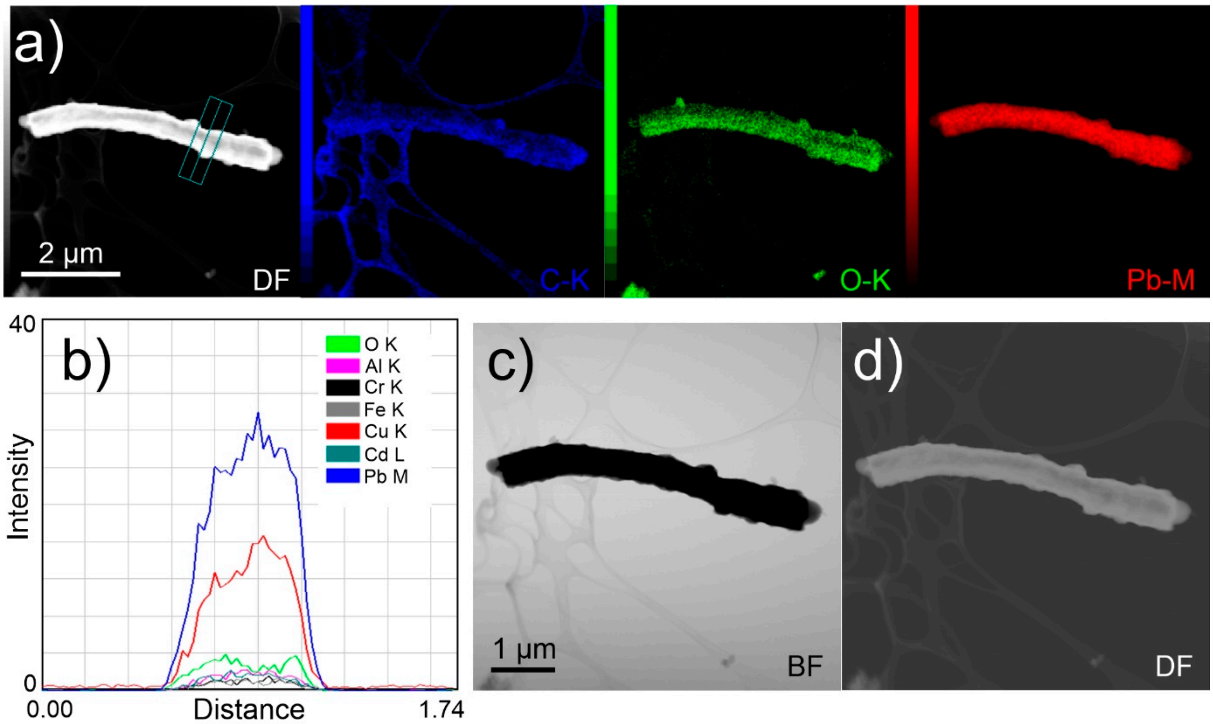

Figure S2. (**a**) scanning transmission electron microscope (STEM) image in darkfield (DF) mode of a Pb whisker fragment and elemental mapping of the whisker for carbon (blue), oxygen (green) and lead (red). An elemental line scan was performed (marked by light blue line and rectangle) with the results presented in (**b**). The Cu is a background signal from the Cu holding grid. (**c**) and (**d**) are images of the same whisker in brightfield (BF) and darkfield, respectively.

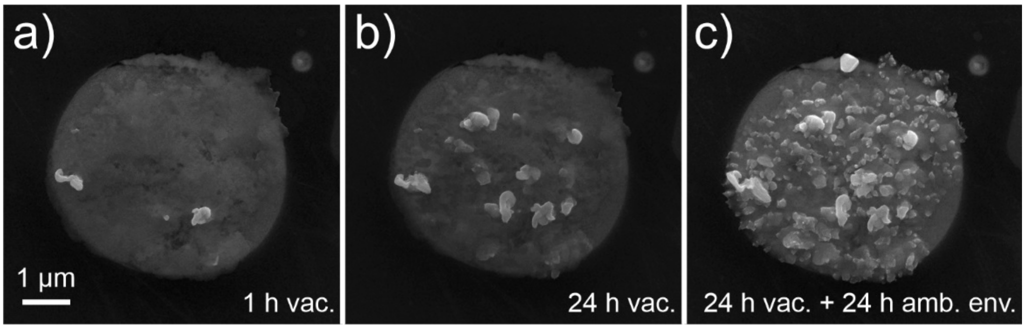

Figure S3. Selected Bi-Mg-Pb pool after (**a**) 1 h in vacuum, (**b**) 24 h in vacuum and (**c**) 24 h in vacuum and 24 h in ambient environment after final sample surface preparation.
